# Does treating with anti–PD-1 to improve glomerular health come without a cost? Reply.

**DOI:** 10.1172/JCI165287

**Published:** 2022-11-15

**Authors:** Stuart J. Shankland, Jeffrey W. Pippin, Oliver Wessely

**Affiliations:** 1 Division of Nephrology, University of Washington, Seattle, Washington, USA.; 2 Lerner Research Institute, Cleveland Clinic Foundation, Cleveland, Ohio, USA.

**Keywords:** Aging, Drug therapy

## The authors reply:

As representatives for our entire team, we thank Jhaveri et al. (1) for their insightful comments on our recent study investigating the increased expression of programmed cell death protein 1 (PD-1) in kidneys during aging and focal segmental glomerulosclerosis (FSGS) (2). In our manuscript, we showed that PD-1 was predominantly increased in podocytes and kidney tubular epithelial cells in both mice and humans. Moreover, in humans, age-elevated glomerular *PCDC1* (gene encoding human PD-1) levels were associated with a lower estimated glomerular filtration rate (eGFR), increased segmental glomerulosclerosis, and reduced arterial intima-to-lumen ratio. We also demonstrated a mechanistic link between increased PD-1 levels in podocytes and their shortened life span. Finally, specifically antagonizing the PD-1 pathway with a specific anti–PD-1 antibody (similar to humanized pembrolizumab or nivolumab) in aged mice and mice with experimental FSGS had major benefits on kidney histology, podocyte life and health span, and tubular epithelial injury (2).

In their response, Jhaveri and colleagues, experts in onco-nephrology, eloquently discuss the clinical kidney-specific adverse events (AEs) when using immune checkpoint inhibitors (ICI) in cancer patients (1). They provide important clinical insights and an up-to-date summary of the incidence and types of glomerular lesions, acute kidney injury, and acute interstitial nephritis observed in patients receiving ICI for cancer treatment (3, 4). Importantly, complete or partial remission of kidney-specific AEs upon discontinuation of ICI treatment in a subset of patients suggests a causal link (3, 4). We unreservedly agree with Jhaveri et al. that caution is warranted when using ICI clinically. In fact, we have *not* advocated the clinical use of anti–PD-1 treatment to limit or reverse kidney aging, nor to be used as a therapy for FSGS.

The clinical data highlighted by Jhaveri et al. underscore the importance of gaining a better understanding of the mechanism or mechanisms underlying kidney complications in patients. While T cell activation, proliferation, and subsequent kidney infiltration are suspected to contribute (3, 4), how this cumulates into kidney dysfunction is unknown. ICIs block the CTLA-4 and/or PD-1 pathways. CTLA-4 acts early in tolerance induction, stopping potentially autoreactive T cells at the initial stage of naive T cell activation, while PD-1 acts late to maintain long-term tolerance, primarily in peripheral tissues (5). Typically a lower incidence of AEs is associated with PD-1 blockade compared with CTLA-4 blockade (3, 4). Interestingly, in our study, mouse *Ctla4* mRNA levels, in contrast with *PD1*, were not elevated in podocytes with age.

There are also several differences between humans and mice that may influence the response to anti–PD-1 treatments. To reconcile these, one needs to experimentally align the animal studies with the therapeutic scenario in human cancer patients. Possible considerations include the following: (a) the duration of therapy — in our study, mice received eight weeks of treatment, while human patients typically receive a 13-week median drug exposure before glomerular disease is first detected; (b) comorbid conditions are oftentimes present in humans (e.g., patients receiving additional medications or already exhibiting altered kidney function before receiving ICI agents), but were absent in our mice; (c) sex and age — the median age of patients developing glomerular disease after ICI treatment is 63 years, and 75% thereof are male, (4) while our mouse study was based on males only; (d) drug dosing — the therapeutic doses of ICIs used in humans might be much higher than the doses of the mouse-specific anti–PD-1 antibody yielding beneficial effects in mouse podocytes; and (e) finally, genetic variation in humans may influence the response to anti–PD-1 treatments, while mouse strains are genetically very homogenous.

We believe that our study has provided some exciting new considerations that have moved us ahead scientifically. First, PD-1 signaling is a new pathway contributing to the aging of podocytes and other kidney epithelial cells as well as the response of podocytes in disease. Second, podocyte aging and disease-induced podocyte injury share a new common pathway — PD-1. This raises the possibility that PD-1 signaling is one of the pathways responsible for the more severe kidney injury when FSGS is superimposed on an aged kidney. Third, the effects of anti–PD-1 antibody treatment are not restricted to the kidney, but also reduced some aspects of liver aging. This suggests that it might be a common aging pathway that needs to be studied further. Fourth, the unexpected discovery of PD-1 signaling in aging leads us to predict that there will be additional surprises in new pathways contributing to kidney aging and disease that will translate into new druggable targets.

## Version 1 09/22/2022

In-Press Preview

## Version 2 11/15/2022

Electronic publication
